# Experiences of patients on cancer treatment regarding decentralization of oncology services at a tertiary hospital in the Eastern Cape

**DOI:** 10.1186/s12885-023-10876-5

**Published:** 2023-05-18

**Authors:** Lumkile Wilmot Jojo, Nonyaniso Trustina Nkutu

**Affiliations:** Faculty of Health Sciences, Department of Public Health, University of Fort Hare, East London, South Africa

**Keywords:** Experiences, Decentralization, Oncology services

## Abstract

**Background:**

The cancer burden is a global public health concern associated with high morbidities and mortalities. Low and middle-income countries are more affected including South Africa. Limited access to oncology services contributes to the late presentation, late diagnosis, and treatment of cancer. In the Eastern Cape, oncology services were previously centralized with negative effects on the quality of life of the already compromised health status of the oncology patients. To mitigate the situation, a new oncology unit was opened to decentralize oncology services in the province. Little is known about the experiences of patients after this transformation. That prompted this inquiry.

**Aim:**

This study aims to explore the experiences of cancer patients regarding the decentralization of oncology services at a tertiary hospital in the Eastern Cape.

**Methodology:**

A qualitative approach with a descriptive, explorative, and contextual design was undertaken, to obtain the perspective of oncology recipients following the decentralization of oncology services at a selected public tertiary hospital in the Eastern Cape. After obtaining ethical clearance and permission to conduct the study, interviews were conducted with 19 participants. All interviews were transcribed verbatim against their audio recordings. Field notes were taken by the primary researcher. The concept of trustworthiness was used to ensure rigour throughout this study. Thematic analysis was done using Tesch’s approach to open coding in qualitative research.

**Results:**

Three themes emerged from the data analysis: 1) Access to oncology services; 2) Oncology services provided; and 3) Need for improved infrastructural facilities.

**Conclusion:**

The majority of patients had positive experiences with the unit. The waiting time was acceptable, and medication was available. Access to services was improved. The staff had a positive attitude towards patients receiving cancer treatment.

**Supplementary Information:**

The online version contains supplementary material available at 10.1186/s12885-023-10876-5.

## Introduction and background

The cancer burden is a global public health concern with an estimated incidence of around 14 million new cases per year, expected to expand to 22 million annually within the next 20 years [10, 24]. Disadvantaged and minority populations suffer worse outcomes compared with white people from Western societies [14, 33]. Without significant interventions in screening and treatment efforts and substantial advances to make cancer services more accessible, the burden of cancer morbidity and mortality is more likely to increase in low- and middle-income countries [21]. The staging of cancer and treatment options play a crucial role in cancer treatment outcomes [30].

Cancer care services are usually costly and centralized, making it difficult for poorly resourced settings to achieve the best possible treatment outcomes. South Africa has embraced a process of decentralization in the transformation of health care and oncology services [12]. Decentralization is meant to bring services closer to the users, thereby making services more accessible to underprivileged communities. However, decentralization has its challenges, and if dealing with those challenges is not effective, managed clients might not reap the full benefits of decentralized healthcare.

Oncology services are also in poor supply in rural areas, particularly in the Eastern Cape Province. The late presentation and late diagnosis may be a result of the limited access to oncology services. In terms of the lack of oncology services, the largely rural communities of the Eastern Cape Province are more impacted than the metropolitan communities. With limited access to oncology care, the Eastern Cape Province, which has a population of over 6,5 million, is severely impacted (Frere Hospital Cancer Registry Report October 2017). Historically, oncology services were primarily provided in East London and Port Elizabeth, which are located in the metropolises of Buffalo City and Nelson Mandela Bay, respectively. Individuals would travel from as far as Lusikisiki to Frere Hospital in East London, a distance of 356 kms, for a week of oncology services.

There has been a need for the decentralization of oncology services in the Eastern Cape, more especially in the eastern region (former Transkei). To reduce the strain on traveling, a decentralized oncology department was built so that cancer patients could get their services closer home without having to sleep on benches halfway before they could be reviewed. It was in 2017 when a functional oncology unit was opened at a tertiary hospital in OR Tambo District Municipality in the Mthatha region. Since the opening of the unit no studies have been conducted to assess how cancer patients feel now that the services had been brought closer to their doorstep. Therefore, this has prompted the primary researcher to conduct this study.

### Aim of the study

The paper aimed to explore and describe the experiences of patients diagnosed with cancer regarding the decentralization of oncology services at a tertiary hospital in the Eastern Cape province of South Africa.

### Research question

- What are the experiences of cancer patients regarding the decentralization of oncology services at a public tertiary hospital in the Eastern Cape Province, South Africa?

## Methods and design

A qualitative research approach was undertaken in this study, to obtain the perspectives and in-depth insights of the oncology patients on the decentralization of oncology services at a public tertiary hospital.

### Population

The population in this study refers to all patients diagnosed with cancer, who were attending an oncology clinic at a selected public tertiary hospital. The population target was both males and female patients ages 35–85 diagnosed with cancer and receiving oncology services at a selected public tertiary hospital from 2017 to 2020.

### Sampling

A non-probability purposive sampling method was used to select participants because they knew the most about their condition and were willing and able to articulate and explain nuances related to their condition [8]. Participants were recruited and selected from the waiting area before they were seen by the doctors. The researcher worked with a nurse in the unit to identify suitable participants. Nineteen clinically stable cancer patients of African ancestry participated in the study. The study site is located in OR Tambo District. The population is predominantly Xhosa. The sample was an aging population from 35 to 85 years old. This study found that majority of participants were older than 50 years. There was only one participant who was between the ages of 41 years and 50 years; one participant was between the ages of 35 years and 40 years whilst another participant was under the age of 35 years. Researchers agree that cancer patients are increasingly getting older [7, 25]. As you get older you are more likely to develop cancer. Old age is the biggest risk factor for cancer. Researchers are not sure what is the reason for this. It could be the fact that elders have been exposed for longer to cancer-causing agents like sunlight, cigarette smoke, and other chemicals [27]. Most of the participants were females. Out of the 19 participants, 18 were female and only one was male. According to the Eastern Cape Cancer Registry report from 2003 to 2007, about 60% of confirmed cancer patients in the province were females. The South African National Cancer Registry report of 2018 shows that 52% of cancer patients in the country are females. Although this study could not delineate the kinds of cancers, researchers concur with these results to say those female cancers in the country are on the rise, especially breast cancer and cervical cancer [23].

### Inclusion criteria

The sample included cancer patients who were managed at a selected public tertiary hospital, both male and female patients diagnosed with cancer, from the ages of 35 years to 85 years old. Only clinically stable patients were included.

### Exclusion criteria

The patients receiving care from private providers and those who were receiving treatment from other public hospitals were excluded. Extremely ill patients and those who were not clinically stable were also excluded.

### Research setting

The research was conducted at the oncology unit of a public tertiary hospital, in OR Tambo Municipality of the Eastern Cape Province of South Africa. The unit is a day clinic without admission beds. Patients who need admission are admitted to medical wards. There is a waiting area with 13 beds for patients with referrals for radiotherapy in East London. The unit has an average of 500 consultations per month, with 100 patients receiving chemotherapy services every month.

### Ethical clearance

Ethical clearance to conduct the study was obtained from the University of Fort Hare ethics review committee (Ref # 2021 = 03 = 07 = JojoL) followed by the acquisition of permission from the Department of Health epidemiological research unit.

### Data collection

Data collection is a process of gathering and measuring information on variables of interest, in a systemic fashion that enables one to answer research questions and evaluate outcomes [11]. In this study, data was collected through semi-structured, one-on-one interviews until the data saturation point was reached. An interview guide with open-ended, semi-structured questions was developed as a research instrument. Ensuring accuracy throughout the process of collecting data and the correctness of the results requires the presence of elements such as credibility, dependability, transferability, and confirmability. Lincoln and Guba’s model was used to promote trustworthiness [28].

### Research instrument

A research instrument is a tool or a method by which a researcher obtains data from the participants for his research project [13]. A self-developed interview guide with open-ended, semi-structured questions was used as a research instrument for data collection. The interview guide had 2 sections namely: Section A which concerned demographic information of the participants and Section B which concerned questions about the experiences of cancer patients who were receiving treatment from the selected public tertiary hospital oncology unit. Section B included questions about the views of oncology patients regarding the quality of services they receive from the unit such as waiting time, availability of medicines, staff attitudes, and accessibility of oncology services since that the new unit had been opened closer to the people of O.R. Tambo District Municipality.

### Data collection procedure

The primary researcher recruited the participants from the waiting area at a selected public tertiary hospital oncology unit. When patients come for their review in the unit, firstly they are registered by clerks, and from there they go to the observation room for vital signs. After checking vital signs, they go to the waiting room before they are seen by the doctors. It was at this stage that the primary researcher recruited the participants after the observations and if they were found to be clinically stable.

The primary researcher was working at the Accident and Emergency Department of the selected public tertiary hospital during the time of the study. The study participants were not his patients. He organized a private room in the oncology unit where interviews were conducted to maintain confidentiality. Data were collected through semi-structured, one-to-one interviews until the data saturation point was reached. A semi-structured list of questions was used to ensure that critical points were covered in every interview. Participants were given the necessary flexibility to enable them to give information on the discussion point relevant to them. That allowed participants to express their experiences regarding the decentralization of oncology services at this public tertiary hospital. The participants were sampled from the waiting room before they were seen by the doctors. The sampled participants were interviewed on a one-to-one basis for about 20 min. All interviews were audio-recorded with permission from the participants and transcribed verbatim in full by the data collector. Every transcript was vigorously checked against its corresponding audio record for accuracy.

### Data analysis

In this study, after the interviews were transcribed, the transcripts were presented to the independent coder along with the methodology chapter, and a copy was made for the primary researcher’s use of the transcribed data.

The coding process consisted of taking the recorded audio data and segmenting these words or paragraphs into themes and sub-themes [9]. Data analysis was done according to Tesch’s eight-step method where the researcher, read each interview, understood the information, and wrote down the thoughts that came to mind. Similar topics were arranged in groups. Then, the researcher coded and wrote the codes next to the appropriate segment of the text. The data was then organized to check if new categories or codes emerged, and all this was done with the assistance of an independent coder. The most descriptive wording for the topics was found and converted into categories. The codes were then arranged alphabetically. A preliminary analysis was performed. Existing material was recorded where necessary [5].

The data analysis progression involved data collection through individual interview discussion, field notes taken by the researcher, transcription of audio recordings, sending transcripts to an independent coder, and the researcher engaging with the data and transcripts to construct themes using Tesch’s steps of data analysis. All codes generated were guided by information from the responses of the patients. The codes were grouped into themes and subthemes.

## Results

Nineteen clinically stable cancer patients of African ancestry participated in the study. The sample was an aging population from 35 to 85 years old. Most of the participants were females. Out of the 19 participants, 18 were female and only one was male (Table 1). Below shows subthemes that emerged from the analysis and quotes from the participants.

**Table 1:** Below shows themes and subthemes that emerged from data analysis

Subthemes	Quotes from the participants
Waiting time	Short waiting time *“I am quickly attended to, 13 min, we do not have to wait that long.” (Participant 4, female, 75 years)* *“Since I do not know how to count, I cannot say the exact time, but it was a few minutes.” (Participant 14, female, 69 years)* *“We do not wait for too long. It is just a while, I do not have to wait that long.” (Participant 15, female, 59 years)* *“I did not stay long; it was not more than 30 min.” (Participant 10, female, 37 years)* *“It depends on the reasons for the visit; if its chemotherapy, I am quickly attended to, but if it is for anything else, it is around 30 min.” (Participant 9, female, 51 years)* *“Over an hour.” (Participant 16, female, 61 years)*
Availability of human resources	Always seen by clinicians on their hospital visits. “*No, it did not happen to me, I always see a doctor*.” (Participant 4, female, 75 years) “*No, I have never left without seeing a doctor*.” (Participant 9, female, 51 years) “*No, it has not yet happened that I go home without seeing a doctor*.” (Participant 12, female, *A*lways get prescribed medications. “*Yes, I always get it [prescribed medications]*.” (Participant 7, male, 70 years) “*Yes, I got them [prescribed medications] all.*” (Participant 9, female, 51 years) “*When I was coming here, I was told I would get them on Monday last week, but first I got them.*” (Participant 11, female, 92) Few participants reported not having their prescribed medication all the time they visited the hospital. “*One time I was seen by nurses, but I was not given the injection I came for. I do not remember the reason why I was not given the injection.*” (Participant 16, female, 61 years) “*I was prescribed medication in East London, where they did not make a prescription.*” (Participant 19, female, 32 years) “*No, I have never been given pills here. It is only that injection.*” (Participant 18, female, 63 years) “*I got the medication in East London.*” (Participant 1, female, 70 years) “*Most of them did not tell me I was on treatment, so they just gave me the pills when I was there; the last time I was given pills was in eMadzikane; here in Mandela, I was not given pills. The doctor checked me and then told me to go.*” (Participant 2, female, 49 years)
The attitude of healthcare workers towards cancer patients	Positive staff attitude, both nurses and doctors were nice and caring. “*I see they are very loving and very caring*.” (Participant 11, female, 92 years) “*Yes, they are nice people; no one has ever shouted at me*.” (Participant 8, female, 52 years) “*The nurses are right people who can talk to people well*.” (Participant 19, female, 32 years) “*They are nice people that you find that I can be thinking about them even when I am home*.” (Participant 13, female, 66 years) “*They are very nice people. One of the nurses gave me R20 and a lift without me asking for it*.” (Participant 12, female, 62 years) Another participant added that she felt comfortable seeking advice from the nurses “*They are nice people, who are easy to talk to or ask about things you are experiencing*.” (Participant 10, female, 37 years) One participant had a negative report about the attitude of nurses “*The doctors are all nice, but there are only nurses who are rude.*” (Participant 9, female,
Appropriate treatment and care	Satisfaction with the services offered to them at the unit. *“I am fine with everything, the nurses and the four doctors I have seen; they are treating me well.” (Participant 17, female, 57 years)* Happy with the quality of services. *“The treatment here is very good compared to the one in Frere.” (Participant 9, female, 51 years)* *“They have good service delivery.” (Participant 7, male, 70 years)* *“They treated me very well; this is my second visit since we were transferred here, and the treatment is good.” (Participant 15, female, 59 years)* High expectations of getting well *“I had hope. I did not think I was going to die; I was hoping that I will be all right. I thought I would be given IV drips; I did not know I was going to be given a pill. I thought when I start chemo, I will be worse or maybe have other sicknesses, but I saw myself getting better.” (Participant 14, female, 69 years)* *“I was expecting to live and to be treated.” (Participant 17, female, 57 years)* *“I did not know what to expect, I went to the doctor, and he said I had cancer, I wanted to live.” (Participant 13, female, 66 years)*
Improved access to services	All the participants appreciated having this facility close to home and receiving cancer treatment. Patients can now access cancer care personnel and services with minimal delays. “*The issue with East London is it will be dated 17 May, and I will meet with a doctor again on 17 June or July and but here it takes 27 days*.” (Participant 17, female, 57 years) “*It used to be a long process because I would have to sleep here, then wake up here going to East London, and when I am coming back, it is still the same process*.” (Participant 10, female, 37 years) “*It has made a big difference. Since we have started being treated here, things have been easy*.” (Participant 12, female, 62 years) “*Coming here is a big difference because here you see a doctor soon/early and go home early. When I go to East London, I stay at the waiting room. It is cold and be transported to East London*.” (Participant 16, female, 61 years) *"Now it takes one day to see a doctor. This place is helpful*.” (Participant 6, female, 55 years) “*Coming here, I go back from on the same day, but the time I was going to East London, I would have to sleep here first before going to East London*.” (Participant 12, female, 62 years) “*It is 2 days when I am going to East London, but it is a day coming here*.” (Participant 17, female, 57 years) “*It was three days for East London; now it is a day*.” (Participant 13, female, 66 years) “*I used to take a week to and from East London; now it is only a day to and from my home*.” (Participant 9, female, 51 years)
Building	overcrowding at the center, fears of contracting Covid19. insecurity, lack of warm water for bathing, lost hospital files, and delayed delivery of laboratory results “*I am happy, but the process is tiring. It would be better if there were beds so that I get the drip in bed.*” (Participant 12, female, 62 years)
Safety and security	*“The water is cold for bathing in the morning for those who sleep here. There is also the issue of security guards. It is not safe where we are sleeping when going to East London. There is a mistake in the list that is made when we get into these ambulances going to East London. Moreover, as there is a Covid, the place where we sleep is congested*.” (Participant 9, female, 51 years) “*The results take longer to come out, and at times they say that your file is lost, they should take note of that*.” (Participant 16, female, 61 years)

## Discussions

### Themes

#### Theme 1: Access to oncology services

The data collected from the participants showed that bringing the oncology services closer to people helped them ease the burden of traveling long distances for treatment. Decentralization of oncology services close to reaching areas improved patient care and positive outcomes, and access to treatment, especially where clients found it difficult to travel to centralized centers [17].

Most of the participants appreciated having this facility close to home and receiving cancer treatment. “*It has made a big difference. Since we have started being treated here, things have been easy*.” (Participant 12, female, 62 years).

“*Coming here is a big difference because here you see a doctor soon/early and go home early. When I go to East London, I stay at the waiting room. It is cold and be transported to East London*.” (Participant 16, female, 61 years).

According to most of the participants, they can now access cancer care personnel and services with minimal delays.

*"Now it takes one day to see a doctor. This place is helpful.”* (Participant 6, female, 55 years).

*“Coming here, I go back from on the same day, but the time I was going to East London, I would have to sleep here first before going to East London.”* (Participant 12, female, 62 years).

*“It was three days for East London; now it is a day.”* (Participant 13, female, 66 years).

Decentralization also led to an increase in access by removing the impediment of traveling a long distance to obtain cancer care services [2]. The study findings are in line with Batho-Pele principles (1997) as far as access to services is concerned. It states that all South African citizens should have access to services to which they are entitled.

##### Subtheme 1.1: Waiting time

In this study, most participants reported that a doctor saw them within a brief period during their last two visits. *“I am quickly attended to, 13 min, we do not have to wait that long.” (Participant 4, female, 75 years).*

Nine participants indicated that they waited for less than 30 min, eight for 30 min to 1 h, and only a few waited for more than 1 h. *“I did not stay long; it was not more than 30 min.” (Participant 10, female, 37 years).*

*“Over an hour.”* (Participant 16, female, 61 years)

According to National Core Standards 2011, acceptable waiting time is up to 3 h for a tertiary hospital. Most patients were seen by the clinicians within an hour which is the acceptable waiting time. Therefore, the decentralization of oncology services in this public tertiary hospital did not compromise the quality of services. World Health Organization identified patient waiting time as a measurement of a responsive health system. In the United Kingdom, according to the patient charter, all patients must be seen within 30 min [32]. A study conducted in Ghana showed that the average waiting time in a public hospital outpatient department is 2 to 3 h before the patient is seen by a doctor [1].

##### Subtheme 1.2: Availability of human resources

All of the participants admitted that they had never been to an oncology unit and had gone home without seeing a doctor. “*No, I have never left without seeing a doctor*.” (Participant 9, female, 51 years).

“*No, it has not yet happened that I go home without seeing a doctor*.” (Participant 12, female, *A*lways get prescribed medications. “*Yes, I always get it [prescribed medications]*.” (Participant 7, male, 70 years).

The study's conclusions are consistent with decentralization's goals and underlying assumptions, which hold smaller firms to higher standards of accountability and responsiveness [3]. Clinical staff had less patients to care for, which increased provider-patient engagement and decreased workload at higher institutions in developed countries, where decentralization of cancer treatment improved patient outcomes [2, 34]. Similar findings are shown by several finished investigations. In cases when patients found it challenging to travel to centralized centers, the study demonstrated that decentralization of cancer services enhanced patient care, access to treatment, and had beneficial outcomes [17]. When compared to centralized services, the idea of building more locally run, locally accountable organizations offers considerable promise [3]. Data gathered indicated that every patient in this decentralized oncology unit was visited by a clinician each day they came for treatment. The unit's services are improved as a result of this. Patients always anticipate seeing a doctor when they visit the hospital for treatment. The quality of the service provided suffers if that does not occur because of crowding or a staffing deficit. South Africa's health system is experiencing a staffing crisis [22]. However, it was also stated that the facility was negatively impacted by issues such as a shortage of radiographers in hospitals, outdated technology, and a heavy patient load of cancer patients [31]. Researchers also concur that the severe oncologist crisis, which has resulted in a significant decline in clinical and radiation oncologists in the university and government sectors, is not an uncommon observation [4].

#### Theme 2: Oncology services provided

All the participants had positive experiences regarding the services provided to them and what they expected from the oncology unit. *“I am fine with everything, the nurses and the four doctors I have seen; they are treating me well.” (Participant 17, female, 57 years).*

*“They treated me very well; this is my second visit since we were transferred here, and the treatment is good.”* (Participant 15, female, 59 years)

These results concur with other studies showing that decentralization of oncology services leads to positive experiences and improved patient outcomes [2, 34]. The decentralization of oncology services to this public tertiary hospital showed improved patient care. This is confirmed by participants that they are always attended to by the healthcare workers during their appointments and the services they get from the facility are satisfactory.

##### Subtheme 2.1: Attitude of health care workers towards patients

Almost all participants described the nurses and doctors as caring and loving people. They experienced positive attitudes from the clinicians during patients' care and personally. “*I see they are very loving and very caring*.” (Participant 11, female, 92 years).

*“Yes, they are nice people; no one has ever shouted at me.”* (Participant 8, female, 52 years).

*“They are nice people, who are easy to talk to or ask about things you are experiencing.”* (Participant 10, female, 37 years).

These optimistic attitudes helped the participants, who were delighted as a result. The results of this study are supported by other investigations. According to these studies' findings [16, 26], staff attitude and knowledge play a significant role in patients' ability to successfully recover from chronic and terminal illnesses.

##### Subtheme 2.2: Appropriate treatment and care

The facility's care and treatment of the participants was deemed satisfactory by most of them. They were content since the care and therapy they were receiving were enabling them to get better. Some of them said that they had never complained and had never left the institution feeling worried. Below are some of the participants answers when they were asked about the treatment, they are receiving whether it was helping them, or it as appropriate for cancer patients. “*I would think so because as I am on treatment here, I am not sick of anything else; they said my hair may fall out, it has not fallen out; they said I will be dizzy, and my color will change*.” (Participant 12, female, 62 years).

“*Yes, I get it…no more pain in the breast.”* (Participant 13, female, 66 years).

*“Yes, I receive everything, and I take it well until it is finished. Yes, I came to do a scan this morning.”* (Participant 7, male, 70 years).

In research done in Honduras, similar impacts of the decentralization of the health system were noted. Making social services more effective for the poor was the study's main objective. The number of services for women's health that are preventative has increased. Additionally, there was a rise in the quantity of patient consultations with doctors and a development in the provision of healthcare services [35]. 15 participants reported that their better health state was proof that they were getting the right treatment and care. The oncology unit at this public tertiary hospital appeared to provide acceptable treatment and care to its patients, in contrast to the South African Human Rights Commission's report detailing how the Department of Health had failed its cancer patients. The report also noted that hospitals were experiencing a lack of chemotherapy medications in addition to a drop in the number of specialists [20]. In the hospital where the study took place there was only one specialist oncologist, but the chemotherapy medications were available.

#### Theme 3: Need for improved infrastructural facilities

These participants were adamant that better infrastructure facilities were required.

##### Subtheme 3.1: Buildings

Participants expressed the need for building structures with more space and wards to accommodate patients that sleep over at the unit. They also mentioned lack of hot water facilities like showers. Below are some of the quotations from the participants. “*I am happy, but the process is tiring. It would be better if there were beds so that I get the drip in bed.*” (Participant 12, female, 62 years).

They expressed concerns about catching Covid-19 while lamenting the overpopulation at the sleepover ward before going to East London. “*Moreover, as there is a Covid, the place where we sleep is congested*.” (Participant 9, female, 51 years).

Shortage of hot water for bathing were also issues brought up. *“The water is cold for bathing in the morning for those who sleep here.”* (Participant 9, female, 51 years).

##### Subtheme 3.2: Safety and security

Safety and security at the unit was also a concern. Below are some of the quotations from the participants.

*“There is also the issue of security guards. It is not safe where we are sleeping when going to East London.”* (Participant 9, female, 51 years).

The study's conclusions are consistent with those of earlier investigations carried out by other researchers [15, 29]. Lack of adequate infrastructure and resources is the main obstacle to cancer screening, diagnosis, and treatment [29]. In low-income nations, a lack of infrastructure frequently presents difficulties [29]. For low-income nations, it is significantly more difficult when it comes to purchase, use, and maintenance of radiotherapy equipment [29]. The provision of oncology services was hampered by a shortage of resources, including qualified healthcare personnel and health infrastructure for the management of cancer, according to a survey conducted in Vietnam among healthcare professionals [15].

### Limitations of the study

Only one hospital was included in the study (selected public tertiary hospital). The study's conclusions might, however, be applied in a different situation. The study was carried out throughout the COVID-19 pandemic. As human interaction was monitored and limited, these were challenging times that proved to be both a challenge and a constraint.

## Conclusion

The findings of the study showed that patients diagnosed with cancer and attending oncology clinics at selected public tertiary hospitals had positive experiences in this decentralized oncology unit. For many of the participants, it appeared as though their expectations had been met by the unit. Their experiences and treatment journey were also made easy by positive staff attitudes. All patients were treated well by the staff; they were also given the necessary support like counseling. They were attended to as soon as possible. This led to shorter waiting times. The availability of medicine was also a noted positive factor in their experience. Even though there were complaints about infrastructure and lack of resources, the overall experiences of patients were positive, and the services rendered were of an acceptable standard.

## Supplementary Information

**Additional file 1.** Interview guide for patients attending oncologyclinic.

## Data Availability

All data is available on request from the corresponding author.
