# Supplementary material for: Experiences of patients on cancer treatment regarding decentralization of oncology services at a tertiary hospital in the Eastern Cape
Source: BMC Cancer. 2023 May 18;23:453. doi: 10.1186/s12885-023-10876-5 (PMC10195123; doi:10.1186/s12885-023-10876-5)
Supplement: Supplementary file 1 — Additional file 1. Interview guide for patients attending oncologyclinic. [file 12885_2023_10876_MOESM1_ESM.docx]

**INTERVIEW GUIDE FOR PATIENTS ATTENDING ONCOLOGY CLINIC**

Please supply information where necessary and tick where appropriate.

**SECTION A**

**SOCIO-DEMOGRAPHIC INFORMATION**

Nurse Medical Doctor Patient Relative/Caregiver of patient

**1**. Age 21-30 31-40 41-50 50 and above

**2**. Sex: Male Female

**3**. Job position (health care providers) ____________________________________

**4.** Months in oncology unit 0-6 months 6-12 months >12 months

**5.** Year diagnosed with cancer (for patients and relatives)

**6.** Number of months receiving therapy in NMAH 0-6 months 6-12 months >12 months

**SECTION B**

**FACTORS AFFECTING ONCOLOGY SERVICES (FOR HEALTH WORKERS ONLY)**

**7.** In your opinion, do think the unit is adequately staffed to offer comprehensive oncology care services?

Please explain______________________________________________________________

**8**. Do you have the necessary equipment that you need to offer functional oncology care services?

Please explain______________________________________________________________

**9.** Do you have the basic infrastructure for oncology care services?

Please explain ______________________________________________________________

**10**. Explain the way you see oncology services in this unit

_______________________________________________________________

**11**. Can you please describe your satisfaction with the functioning of the oncology unit in this unit?

Please explain_______________________________________________________________

**12**. Is there anything you would like to recommend for the improvement of oncology care services in this hospital?

Please explain_______________________________________________________________

**SECTION C**

**QUALITY OF ONCOLOGY SERVICES (For patient and relatives of patients only)**

**13.** How often do you visit the facility to receive cancer care services?

Once a month two or more times a month

**14**. In your previous two visits, how long did you had to wait to be seen by a Dr?

≤ 30 minutes 30 minutes-1 hour > 1-2 hours > 2 hours

Please explain_________________________________________________________________

**15**. In your previous two visits, did you receive all prescribed medication for your treatment?

Please explain_________________________________________________________________

**16** What is the staff attitude of nurses and Doctors at this oncology unit?

Please explain_________________________________________________________________

____________________________________________________________________________

**17**. Are there any other comments you would like to make about the cancer-care services at this hospital? _______________________________________________________________________

This is the end of the questionnaire – thank you very much for your time.
